# Protective role of heparin in the injury of the liver and kidney on the experimental model of ischemia/reperfusion

**DOI:** 10.1186/1749-8090-9-35

**Published:** 2014-02-17

**Authors:** Ali Ümit Yener, Mustafa Cüneyt Çiçek, Serhat Bahadır Genç, Turgut Özkan, Emre Doğan, Bülent Çağlar Bilgin, Tezcan Akın, Havva Erdem, Handan Ankarali

**Affiliations:** 1 Department of Cardiovascular Surgery, Ankara Numune Education and Research Hospital, 06330 Ankara, Turkey; 2 Department of Cardiovascular Surgery, Nevsehir State Hospital, 50200 Nevsehir, Turkey; 3 Department of Cardiovascular Surgery, Ahi Evren Education and Research Hospital, 61040 Trabzon, Turkey; 4 Department of General Surgery, Ankara Numune Education and Research Hospital, 06330 Ankara, Turkey; 5 Department of Histopathology, Medical Faculty, Duzce University, Konuralp, Duzce, Turkey; 6 Department of Biostatistics, Medical Faculty, Duzce University, Konuralp, Duzce, Turkey; 7 Department of Cardiovascular Surgery, Çanakkale Onsekiz Mart University, Kepez, Çanakkale, Turkey

**Keywords:** Heparin, Ischemia, Reperfusion, Lung, Kidney

## Abstract

**Background:**

Surgery of thoracoabdominal aortic aneurysms (TAAA) is associated with high incidence of serious complications. Ischemia/reperfusion (I/R) injury may be responsible for these complications. We investigated the effect of degree of anticoagulation on remote organ I/R injuries and whether heparin is protective against I/R injury in addition to its anticoagulant properties.

**Methods:**

Spraque Dawley rats were used to determine both liver and kidney concentrations of HSP-70,IL-6, MPO in four groups: ischemic control (operation with cross-clamping and intraperitoneal administration of 0.9% saline, n = 7), sham (operation without cross-clamping, n = 7), heparin (ACT level about 200), and high dose heparin (ACT level up to 600). Histological analyses of the organs were performed.

**Results:**

Histopathological evaluation of kidney presented significant differences between groups with regards to the cytoplasmic vacuole formation, hemorrhage, tubular cell degeneration and tubular dilatation while heparinized group had best results. The kidney MPO and HSP-70 levels significantly decreased (p < 0.05), but IL-6 level was not significant (p > 0.05) in heparinized group when compared to ischemic control group. No statistically significant intergroup differences were detected in the tissue samples of liver. Immunohistochemical markers of the liver were compared and no statistically significant difference was found among the groups.

**Conclusion:**

Heparin is an important anticoagulation agent in TAAA surgical procedures but the use of higher levels of heparin in the present study revealed no beneficial effects. Bleeding complications is much less when heparin is used in the real-world clinical practice as ACT levels of 200.

## Background

Surgical repair of thoracoabdominal aortic aneurysms (TAAAs) are highly complex and challenging interventions. It is not surprising that this operation is associated with a high incidence of serious complications, often leading to respiratory failure, renal failure, neurological deficits or even death.

Multiorgan dysfunction, presumably related to activation of inflammatory pathways and over production of cytokines and other inflammatory mediators, is a major cause of death. The systemic inflammatory response syndrome (SIRS), ischemia-reperfusion injury (I/Ri), and inflammatory pathway activation are considered precursors of morbidity and mortality after open TAAA repair [1-3].

Cytokines such as IL-6 are important mediators of the inflammatory response in ischemia. Plasma IL-6 concentration increased gradually, reached its peak 24 hours after surgery, and correlated with aortic cross-clamp time and morbidity. The observed correlation between plasma IL-6 and aortic cross-clamp time supports the hypothesis that the IL-6 response mainly reflects the impact of ischemia and reperfusion rather than the impact of surgical trauma in elective TAAA surgery. The strong correlation between plasma IL-6 and morbidity in previous reports suggesting that plasma IL-6 is a reliable predictor of outcome in elective TAAA repair [4-6].

Heat shock proteins (HSPs) are cellular stress proteins which have been shown to have an important role for the survival of cells under stress conditions [7]. Zhang et al. [8] pointed out that, HSP70 could respond to a wide variety of stress conditions such as ischemia, and inflammation. It can prevent the irreversible denaturation of proteins [9]. Elevated expression of HSP70 can prevent cell death processes [10]. It has been showed that overexpression of HSP70 attenuate the release of inflammatory factors and interferes with the process of apoptotic cell death [10-12].

Myeloperoxidase (MPO) is one of the obvious indicators for the tissue infiltration of neutrophilic granulocytes. MPO activity increases in response to the I/R injury [13].

Heparin continues to dominate anticoagulation therapy for surgical repair of TAAA and other cardiovascular surgical procedures. Heparin is best recognized for its ability to prevent blood coagulation by catalytically accelerating the interaction of antithrombin III (AT III) with thrombin, as well as with factors XIIa, IXa, Vlla, and Xa, thereby inhibiting the proteases necessary for completion of the coagulation cascade [14].

Beside heparin is known to possess effects independent of its anticoagulant activity, it plays a role in inhibition of leukocyte-mediated damage [15,16]. Other important and often unrecognized actions of heparin and other glycosaminoglycans are their ability to inhibit complement activation [17] and to exhibit anti-inflammatory effects [18-20].

In this study, we investigated the effect of the degree of anticoagulation on remote organ I/R injury and whether heparin is protective against I/R injury beside its anticoagulant properties.

## Methods

### Animal and surgical procedure

This study was approved by the Animal Experiment Committee of Düzce University Graduate School of Medicine and all animal care and use were in accordance with the European Convention for Animal Care. 28 male Sprague–Dawley rats weighing 250 to 350 g were used for the experiment. Rats were fed standard laboratory diets and were maintained in a temperature- and photoperiod-controlled (12 hr/day) room. The rats were followed for 15 days before the procedure. None of the animals had any abnormality before operation.

### Study groups

The animals were randomly divided into four groups (7 rats each). 1) Sham-operation group. The operation was performed in the same form, but without aortic occlusion and heparin administration. 2) Non- heparinized control group rats’ abdominal aortas were clamped for 45 minutes. 3) Low-dose heparin treated group animals’ aortas were cross-clamped for 45 minutes and 400 IU/kg of heparin was administered. ACT was kept around 200 during the procedure. 4) High-dose heparin treated group. In this group rats’ aortas was cross-clamped for 45 minutes and 800 IU/kg of heparin was administered. ACT was kept around 600 during the procedure.

### Operative procedure and technique

Premedication was intraperitoneally applied with ketamine (50 mg/kg) and xylazine (5 mg/kg) to rats. Anesthesia was continued by intermittent injections of ketamine, without endotracheal intubation and mechanical ventilation. Temperature probe was placed in the rectum.

Rats were placed in supine position. After sterile preparation of the surgical area, a standard midline laparotomy incision was made, and after the intestines were retracted the abdominal aorta was explored transperitoneally.The abdominal aorta at the below renal arteries and above the iliac bifurcation levels was cross-clamped during surgical procedures. The duration of the ischemic insult was 45 minutes. Temperature was maintained between 36.5 and 37.5°C during the procedure. Following removal of the aortic clamps, abdominal wall was repaired by using 5/0 polyprolene suture. Penthobarbital (20 mmg/kg) was used as anesthetic at the 48th hour and all the animals were sacrificed. The liver and kidney was dissected totally and fixed in buffered formalin for 7 days.

### Histopahological assessment

The liver and kidney were removed and fixed in 10% formalin for histopathologic examination. About 4 μm thick paraffin sections were cut and collected on glass slides. 5 μm thick sections were located on polylysine-coated slides and were stained with hematoxylin and eosin (H & E). The glass slides were examined by light microscopy (Olympus BX51; Olympus Corp. Tokyo, Japan) at 400× magnification.

### Immunohistochemical assessment

Blood samples were obtained directly from the cardiac of groups of four rats at the end of 48 h immediately before the sacrification. Blood samples were obtained with sterile 10 ml syringe after the chest wall was cleansed with cholorohexidine in spirit. Blood samples for cytokine assay were collected into heparinized (20 unit/ml blood) sterile tubes and immediately transferred on ice to be centrifuged at 2000 rpm (at 4°C) for 10 minutes and stored (-70°C) until the time of assay for IL-6, MPO and HSP-70. These marker levels were obtained from blood samples together with kidney and liver tissue.

### Analysis of HSP

4 μm thick paraffin sections were prepared. Deparaffinization and hydration of tissue sections was performed in xylenes and graded alcohol. The sections were incubated with primary anti-HSP70 (clone BRM.22, dilution 1/80, Biogenex, San Ramon, California) diluted in buffer. Negative control was PBS.

### Analysis of IL-6

Immunohistochemical detection of IL-6 receptor was performed with polyclonal anti-human IL-6 receptor antibody C-20 (Santa Cruz Biotechnologies, Santa Cruz, CA, USA). The antibody was diluted 1: 20. Streptavidin-biotin-peroxidase protocol was applied in IL-6 receptor immunostaining. The secondary anti-rabbit antibody was diluted 1: 500. After the first antibody was omitted negative controls were conducted.

### Analysis of MPO

Immuno-histochemical evaluation of MPO activities of the liver and kidney tissues was performed with an anti-MPO kit according to the manufacturer’s protocol. Deparaffinization and hydration of samples on polylysine-coated slides was performed. Then, the microwave antigen retrieval procedure was performed, and the samples were incubated in a 3% H2O2 solution to inhibit endogenous peroxidase. The sections were incubated with a blocking solution for blocking nonspecific background staining. Then incubation of the sections with primary anti-MPO antibody and with biotinylated goat anti-mouse antibody followed. After incubation with chromogenic substrate (DAB), the sections were counterstained with hematoxylin and eosin (H & E). The slides were examined under a light microscope and two pathologists who did all analyses were blinded to group assignments. The staining of cytoplasmic MPO in the neutrophils was evaluated, and the results were expressed as the percentage of neutrophils cytoplasmically stained positive for MPO. Tissues with no evidence of staining, or only rare scattered positive cells, less than 3%, were recorded as negative. The immunohistochemical results were evaluated for intensity and frequency of staining. The intensity of staining was graded as 0 (negative), 1 (weak), 2 (moderate), and 3 (strong). The frequency was graded from 0 to 4 by the percentage of positive cells as follows: grade 0, <3%; grade 1, 3-25%; grade 2, 25-50%; grade 3, 50-75%; grade 4, more than 75%. The index score was the product of multiplication of the intensity and frequency grades, which was then classified into a 4 point scale: index score 0 = product of 0, index score 1 = products 1 and 2, index score 2 = products 3 and 4, index score 3 = products 6 through 12.

### Statistical analysis

Statistical analysis and calculations were performed with SPSS 15 for Windows (Chicago, IL). Results were expressed as the mean (standard error mean). Intergroup differences are detected by Kruskal-Wallis analysis of variance and Mann–Whitney U test was used for statistical comparisons. While p value of < .01 was accepted as statistically significant p value of < .05 was accepted as statistically less significant.

## Results

There was no significant difference with regards to body temperature, mean arterial blood pressure and heart rate among the groups.

Histopathological evaluation of the tissue samples taken from the kidney showed significant intergroup differences in terms of the cytoplasmic vacuole formation, hemorrhage, tubular cell degeneration and tubular dilatation (P = 0.031, P = 0.003, P = 0.007, P = 0.011 respectively).

There was no cytoplasmic vacuole formation in the kidney specimens of high dose-heparinized group, while cytoplasmic vacuole formation was evident in the other animals’ kidney; as shown in Table 1.

**Table 1:** Histopathologic results of the kidney tissue

**H&E**	**Grade**	**Ischemic control**		**Sham**		**Heparinized**		**High dose-heparinized**		***P values***
		**Number**	**%**	**Number**	**%**	**Number**	**%**	**Number**	**%**	
Cytoplasmic vacuole formation	0	0	0	1	20	4	66.7	3	100	0.031
	1	3	100	4	80	2	33.3	0	0	
Hemorrhage	0	0	0	3	60	6	100	2	66.7	0.003
	1	3	100	2	40	0	0	1	33.3	
Tubular cell degeneration	0	0	0	4	80	5	83.3	3	100	0.007
	1	3	100	1	20	1	16.7	0	0	
Tubular dilatation	0	0	0	5	100	6	100	1	33.3	0.011
	1	1	33.3	0	0	0	0	2	66.7	
	2	2	66.7	0	0	0	0	0	0	

As depicted in Table 2, heparinized group of the examination compared with other groups showed no hemorrhage according to histopathologic evaluation. Hemorrhage was worst in the ischemic control group followed by sham and high dose-heparinized group. Hemorrhage in the kidney specimens of high dose-heparinized group may be due to high levels of ACT (Table 1).

Examination of the kidney tissue revealed that ischemic control group showed most tubular cell degeneration according to histopathologic evaluation (P = 0.007); whereas there was no degeneration in the high dose-heparinized group.

There was no tubular dilatation in the kidney specimens of sham and heparinized groups while tubular dilatation was worst in the ischemic control group (P = 0.011).

Immunohistochemical markers as, MPO, HSP-70 and IL-6 parameters were studied from the kidney tissue samples. While there was no intergroup difference with regards to IL-6 stain (p = 0.205), there were statistical significant differences among the groups with regards to MPO and HSP-70 stains (p value 0.01 and 0.004, respectively) (Table 2). Grade 1, 2 and 3 staining with MPO was strongest in the ischemic control group and high dose-heparinized group whereas there was no staining in the heparinized group (P = 0.01). The lowest HSP-70 level was measured in the sham group, after heparinized groups while the highest HSP-70 level was measured in the high dose-heparinized and ischemic control group. IL6 staining results are given in Table 2. There was no significant difference found among the groups (*P = 0.205*).

**Table 2:** Immunohistochemical results of the kidney tissue

	**Grade**	**Ischemic control**		**Sham**		**Heparinized**		**High dose-heparinized**		***P value***
		**Number**	**%**	**Number**	**%**	**Number**	**%**	**Number**	**%**	
MPO	0	0	0	3	60	3	100	0	0	0.01
	1	3	37.5	2	40	0	0	1	33.3	
	2	3	37.5	0	0	0	0	0	0	
	3	2	25	0	0	0	0	2	66.7	
HSP70	0	0	0	2	66.7	2	40	0	0	0.004
	1	1	12.5	0	0	1	20	0	0	
	2	1	12.5	1	33.3	2	40	0	0	
	3	6	75	0	0	0	0	3	100	
IL-6	0	3	100	2	40	5	62.5	2	66.7	0.205
	1	0	0	3	60	3	37.5	1	33.3	
	2	0	0	0	0	0	0	0	0	

Histopathological evaluation of the tissue samples taken from the liver showed no statistically significant intergroup differences in terms of the hepatocyte degeneration, hepatocyte steatosis, single cell necrosis, inflammatory cell, sinusoidal dilatation and periportal bridging necrosis (P = 0.587, P = 0.277, and P = 0.196 respectively) (Table 3).

**Table 3:** Histopathological results of the liver tissue

	**Grade**	**Ischemic control**		**Sham**		**Heparinized**		**High dose-heparinized**		***P value***
		**Number**	**%**	**Number**	**%**	**Number**	**%**	**Number**	**%**	
Hepatocyte degeneration	1	1	33.3	0	0	2	28.6	-	-	0.085
	2	2	66.7	4	66.7	3	42.9	-	-	
	3	0	0	2	33.3	2	28.6	-	-	
Hepatocyte steatosis	0	2	50	5	100	7	100	-	-	0.116
	1	2	50	0	0	0	0	-	-	
Single cell necrosis	0	1	33.3	4	80	2	28.6	-	-	0.273
	1	1	33.3	1	20	5	71.4	-	-	
	2	1	33.3	0	0	0	0	-	-	
Inflammatory cell	0	1	33.3	2	40	2	28.6	0	0	0.587
	1	2	66.7	3	60	4	57.1	2	66.7	
	2	0	0	0	0	1	14.3	1	33.3	

Comparison of immunohistochemical markers of the ischemia- reperfusion injury of the liver tissue in an aortic occlusion model of rats revealed that there was no statistically significant difference found among the groups in terms of MPO, HSP70 and IL-6 (P = 0.152, P = 0.053, and P = 0.243 respectively) (Table 4).

**Table 4:** Immunohistochemical results of the liver tissue

	**Grade**	**Ischemic control**		**Sham**		**Heparinized**		**High dose-heparinized**		***P value***
		**Number**	**%**	**Number**	**%**	**Number**	**%**	**Number**	**%**	
MPO	0	1	33.3	5	100	3	60	2	66.7	0.152
	1	2	66.7	0	0	2	40	1	33.3	
	2	0	0	0	0	0	0	0	0	
	3	0	0	0	0	0	0	0	0	
HSP70	0	0	0	4	80	2	66.7	1	33.3	0.053
	1	2	28.6	1	20	1	33.3	2	66.7	
	2	3	42.9	0	0	0	0	0	0	
	3	2	28.6	0	0	0	0	0	0	
IL-6	0	1	33.3	0	0	6	85.7	2	66.7	0.243
	1	2	66.7	4	80	1	14.3	1	33.3	
	2	0	0	1	20	0	0	0	0	
	3	0	0	0	0	0	0	0	0	

## Discussion

Despite contemporary approaches to organ protection, open abdominal aortic aneurysm (AAA) repair is still associated with significant morbidity and mortality in high-risk patients [21]. Multiple organ failure (i.e., the lung, kidney) and spinal cord injury are related with activation of inflammatory pathways and over production of other inflammatory mediators during the thoracoabdominal aortic surgery [22,23]. Thoracoabdominal aortic clamping can lead to systemic inflammatory response and ischemia occurs as the blood flow through the major arteries supplying blood to remote organs slows down or stops [24,25].

Among various factors that has been related with end organ ischemia/reperfusion injury such as distal aortic hypotension after aortic occlusion, duration of ischemia and aneurysm extent and microthrombus formation are most important and avoidable factors [26]. So microthrombus formation associated with blood flow slowing, stopping and inadequate heparin dose can cause multiorgan disorders together with histopathological and functional changes [27,28]. Especially, when the hematocrit level is higher than 40 mg/dl microthrombus may easily form. It was suggested that microcirculatory disturbances such as higher blood viscosity characterized by hemoconcentration and microthrombus formation were associated with ischemia [29].

Heparin still remains the leading drug in the management of anticoagulation. Not only heparin has anticoagulant activity but also it has direct anti-inflamatory action. The role of heparin as an anti-inflamatory and anticoagulant agent has a wide spectrum of interactions with various enzymes, hormones, biogenic amines, and plasma proteins. There is great variability in plasma concentration of heparin in relation to dose, so the anticoagulant response is not linear. To provide equilibrium between preventing coagulation and in the mean time preventing excessive bleeding is difficult. This difficulty has increased the importance of the identification of the level of anticoagulation. The most popular and predominant test to monitor anticoagulation is ACT, so we thought to investigate the effect of ACT levels on end organ ischemia in an experimental model.

The presence of patients with postoperative organ dysfunction relationships with response of plasma inflammatory mediators after I/Ri. The overexpression and release of inflammatory mediators, cytokines, activation of phospholipase A2 and complement system, effects of adhesion molecules, activation of arachidonic acid system make I/R injury very complicated and difficult to understand [30-32]. Actually, ischemia and reperfusion is a chain reaction resulting in free oxygen radical generation, respiratory burst of activated neutrophils that occurs in response to tissue injury and the autooxidation of cathecolamines [8]. Severin et al. [33] reported that heparin has anti-inflammatory properties beside its anti-coagulant effects. This characteristic of heparin has been attributed to binding certain cytokines, like chemokines which mediate inflammation through their control of leukocyte migration and activation. We investigated the levels of IL-6, MPO and HSP-70 to evaluate the inflammatory response to I/R injury and the effect of heparin on it.

Acute phase proteins, myeloperoxidase and IL-6 are increase with inflammation [5,34]. HSP-70, a well known cytoprotective agent increases after I/R and its level is directly correlated with the degree of tissue injury and inflammation. In the present study ,the lowest HSP-70 and MPO level in kidney tissue was measured in the sham group and heparinized groups, showed that the inflammation and injury was significantly lower with this treatment. The decrease in HSP-70 in these groups also was an indirect proof of lower tissue injury and better cellular protection. We could not find a significant difference between the groups in terms of the degree of inflammatory response, degree of IL6 kidney tissue.

Cytoplasmic vacuole formation, hemorrhage, tubular cell degeneration and tubular dilatation in the kidney tissue after I/R were decreased after heparin treatment and the histopathologic protective effect of heparin was shown in the present study.

Comparison of immunohistochemical markers and histopathological evaluation of the ischemia- reperfusion injury of the liver tissue in an aortic occlusion model of rats revealed that there was no statistically significant difference. The reason for this outcome for us that; the afferent blood supply to the liver arises from two sources: (i) the hepatic artery, which carries oxygenated blood and accounts for approximately 25% of hepatic blood flow and 50% of total oxygen, and (ii) the portal vein, which drains the splanchnic circulation and accounts for approximately 75% of hepatic blood flow. Because of its protection by a dual blood supply and the capacity for anaerobic metabolism of glycogen stored in the liver, hypoxic damage can occur later in this organ [35].

The half-life of heparin is approximately 30 minutes, 60 minutes and 150 minutes respectively following IV bolus of 25 U/kg, 100 U/kg and 400 U/kg [36]. Our clinical routine in abdominal aortic surgery is to administer bolus of 100 U/kg heparin and to maintain ACT level around 200 seconds. In the present study there was no significant difference between heparin groups (ACT: 200 and ACT: 600) in terms of histopathologic changes or biochemical inflammatory response in the liver and kidney. Also, keeping ACT level around 200 sec during thoracoabdominal surgery seems both to ensure the adequate level of anticoagulation and to avoid the adverse effects such as bleeding.

## Conclusions

Many current analysis clearly evidences that the surgical repair of TAAA remains a challenge even in this century. Recent major progress in our understanding of the pathophysiology and operative strategy have decreased the risk of open TAAA repair complications but obviously further investigation is necessary. Heparin is an important anticoagulation agent in thoracoabdominal and abdominal surgical procedures but no beneficial effects were seen with the use of higher levels of heparin in the present study. Taking into account that, bleeding complications will be much less when heparin used in the real-world clinical practice as ACT levels of 200. Furthermore, the results of this investigation indicate that heparin molecule has provide to potential anti-inflammatory property and renal protective action.

## Abbreviations

TAAA: Thoracoabdominal aortic aneurysm; IL-6: Interleukin-6; MPO: Myeloperoxidase; HSP-70: Heat shock protein 70; ACT: Activated clotting time; SIRS: Systemic inflammatory response syndrome; AT III: Antithrombin III; I/R: Ischemia reperfusion; I/Ri: Ischemia reperfusion injury; H & E: Hematoxylin and eosin.

## Competing interests

The authors declare that they have no competing interests.

## Authors’ contributions

AÜY and MCÇ carried out the design and conduction of the study. SBG, BÇB and ED participated in the design of the study. HE carried out the histopathological examination. HA and TA performed the statistical analysis. TÖ revised and improved the last version of the text. All authors read and approved the final manuscript.
